# Long-Term Exposure to Isoflavones Alters the Hormonal Steroid Homeostasis-Impairing Reproductive Function in Adult Male Wistar Rats

**DOI:** 10.3390/nu15051261

**Published:** 2023-03-02

**Authors:** Sara Caceres, Belén Crespo, Angela Alonso-Diez, Paloma Jimena de Andrés, Pilar Millan, Gema Silván, María José Illera, Juan Carlos Illera

**Affiliations:** 1 Department of Physiology, Veterinary Medicine School, Complutense University of Madrid, 28040 Madrid, Spain; 2 Department of Animal Medicine, Surgery and Pathology, Veterinary Medicine School, Complutense University of Madrid, 28040 Madrid, Spain

**Keywords:** isoflavones, testis, steroid hormones

## Abstract

The consumption of isoflavones is gaining popularity worldwide due to their beneficial effects on health. However, isoflavones are considered to be endocrine disruptors and cause deleterious effects on hormone-sensitive organs, especially in males. Therefore, this study aimed to determine if a continuous and prolonged exposure to isoflavones in adult males altered the endocrine axis effect of testicular function. For this purpose, seventy-five adult male rats were administered with low and high mixtures of isoflavones (genistein and daidzein) for 5 months. The determination of steroid hormones (progesterone, androstenedione, dehydroepiandrosterone, testosterone, dihydrotestosterone, 17β-estradiol, and estrone sulphate) was carried out in serum and testicular homogenate samples. Sperm quality parameters and testicular histology were also determined. The results revealed that low and high doses of isoflavones promote a hormonal imbalance in androgen and estrogen production, resulting in a decrease in circulating and testicular androgen levels and an increase in estrogen levels. These results are associated with a reduction in the sperm quality parameters and a reduction in the testicular weight, both in the diameter of the seminiferous tubules and the height of the germinal epithelium. Altogether, these results suggest that a continuous exposure to isoflavones in adult male rats causes a hormonal imbalance in the testes that disrupts the endocrine axis, causing defects in testicular function.

## 1. Introduction

It is well established that exposure to endocrine disruptors can suppose a health risk [1]. These molecules act similarly to endogenous hormones, causing alterations to the metabolism. Isoflavones are considered to be endocrine disruptors since their structure, similar to 17b-estradiol, gives them highly estrogenic properties that affect the reproductive tract, among other organs, and because they are naturally found in plants such as soybeans [2]. On the other hand, several authors associate isoflavones’ effects with a considerable number of benefits [3] that include protection against breast or prostate cancer, a reduction in the incidence of cardiovascular disease, or a relief of menopausal symptoms [4]. A particular benefit that is gaining relevance is the effect of isoflavones on adipogenesis regulation, making them able to reduce body weight [3,5].

Due to all of these beneficial and nutritional properties, soy-based products are rising in popularity, leading to an increase in their consumption worldwide, from the infant to elder populations. Interestingly, the United States Department of Agriculture (USDA) has included the use of soy-based products as an alternative to animal proteins [6,7]. It must be considered that soy-based products that are consumed daily, such as soymilk, soy-yogurts, or infant formulas, contain a range of isoflavones between 25 and 190 mg/100 g [6], which could turn into a continuous and elevated isoflavone intake.

On the other hand, the endocrine axis function is influenced by environmental signals and the developmental period. These factors alter the gene expression and cell signaling that permit the organism to adapt and develop [2]. As isoflavones have the capacity to interfere with the endocrine axis function, their consumption can lead to changes in the cell signaling pathways that are associated with hormonal homeostasis. Therefore, the consumption of isoflavones may cause a deleterious effect on the organism. Most studies have been carried out on females, due to the estrogenic activity of isoflavones. However, the endocrine axis function is different in males and females and, therefore, isoflavones may alter the endocrine axis in a different manner. Indeed, several studies have affirmed that isoflavone consumption produces reproductive disorders in both males and females [2,8,9,10,11]. In women, supplementation with isoflavones was found to affect endocrine profiles, causing alterations to their menstrual cycles [8] and menstrual regularity [11]. Regarding men, recent studies have reported that isoflavone intake has an impact on testicular function and sperm quality [2,10]. Moreover, it has been shown that these disorders in testicular functions may be product of hormonal changes caused by isoflavone consumption. In prepuberal male rats, an administration of low doses of isoflavones leads to alterations in androgen and estrogen serum and testicular levels, which triggers a delay in the onset of puberty.

Furthermore, the rats that were fed with high doses of isoflavones did not reach puberty during the time of experiment [9,12]. Other authors also associated these changes in hormonal levels with testicular histological changes, deficiencies in spermatogenesis, and a reduced content of spermatozoa [10,13,14]. However, these studies differ with regard to the gender and the age of the animal used, the dose of isoflavones, the administration route, and the time of the exposure to the isoflavones. Most of the studies carried out on males were developed with a short-term exposure to isoflavones and during early periods of their development, where the endocrine axis plays a critical role, and minimal changes can alter the axis’ regulation. Therefore, it can be assumed that exposure to phytoestrogens during periods such as puberty may exert an effect on the endocrine axis. However, few studies have considered the effects of a long-term exposure to isoflavones in adult males. Most of the studies carried out on adult males administrated the isoflavones for a short period of time, showing that isoflavones cause several dysfunctions [14,15].

Assuming that isoflavones cause a dysregulation in testicular function, this study aims to elucidate if a long-term exposure to isoflavones can cause deleterious effects on the endocrine axis, as well as induce testicular changes in adult intact male rats that are not exposed to any endocrine changes related to development. For this purpose, adult male rats were administered with low and high doses of isoflavones for 5 months, and their steroid hormone profiles and sperm quality parameters were determined.

## 2. Materials and Methods

### 2.1. Animals

A total of seventy-five 60-day old male Wistar rats (RjHan: WI, Janvier Labs, Madrid, Spain), weighing 306.52 ± 12.71 g at the beginning of the experiment, were used. In order to standardize the experimental conditions, the choice of this strain of rats was considered after previous studies on this same strain, as previously detailed [9,12,13]. The rats were housed in Meraclon cages, with dimensions of 25 cm × 47.5 cm × 20 cm, divided in groups of five animals per cage, and maintained in a temperature-, humidity-, and light-controlled room: 20 ± 2 °C; 45% relative humidity; and 12-h light:12-h dark cycle (from 08:00 a.m. to 08:00 p.m.). All the rats were fed with a standard laboratory pellet commercial diet (Sodispan S.L., Madrid, Spain) and water ad libitum. Their absolute food and water consumption was measured weekly in all the cages, as previously reported [16]. Briefly, their food and water consumption was determined by subtracting the leftover volumes/weights from the initial volumes/weights. Their food consumption was expressed as g/rat/week and their water consumption as ml/rat/week. The required sample size that was needed to simultaneously compare the normal means of the three experimental groups (control plus two treatments) was obtained using the sample size determination module of the statistical package Statgraphics Centurion XVI (Statpoint Technologies, Inc., Warrenton, VA, USA). The experimental protocols adhered to the guidelines of the Council of European Union, and were approved by the Institutional Animal Care and Use Committee of the University Complutense of Madrid, and the Animal Protection Area of the Community of Madrid, Spain (Ref: PROEX 175/19).

### 2.2. Dietary Treatment

The isoflavones were administered orally over a period of 5 months (20 weeks). The selection of the doses was based on previous studies [9,12]. The rats were randomly assigned to three groups of 25 rats each: control, low mixture of isoflavones (17 mg kg^−1^ day^−1^ genistein + 12 mg kg^−1^ day^−1^ daidzein), and high mixture of isoflavones (170 mg kg^−1^ day^−1^ genistein + 120 mg kg^−1^ day^−1^ daidzein) (LC Laboratories, Woburn, MA, USA). The isoflavone mixture was administered by dissolving them in drinking water at the studied concentrations, allowing for a voluntary consumption by the animal. In order to provide the animals with the correct isoflavone dilutions, two weeks before carrying out the experiment, the water consumption was measured daily in all the cages to estimate the average water consumption. Every four days, the drinking water was replaced to provide the animals with fresh isoflavone dilutions. For the control group, drinking water without isoflavones was administered.

Data from the rat’s body weights were collected each week, in order to observe the effect of the isoflavones in this parameter, and to re-calculate the isoflavone dilutions. Every 4 weeks, five animals from each group were sacrificed by cervical dislocation. Prior to sacrifice, the animals were anesthetized, and their blood samples were collected.

### 2.3. Serum Samples

Blood collection was performed randomly on five animals of each group every 15 days, from the dorsal aorta under anesthesia, and prior to sacrifice via cardiac puncture. The animals were anesthetized with isoflurane (IsoVet 1000 mg/g, B Braun VetCare SA, Barcelona, Spain) at 4% for induction and 1.5% for the maintenance of the supplies, in a fresh gas flow rate of 0.6 L of oxygen/min. The blood samples were centrifuged at 1200× *g* for 20 min at 4 °C. The serum was separated and stored at −20 °C until the hormonal analysis.

### 2.4. Testes Homogenates and Histology

Testes were collected for homogenates and histology at necropsy, and the testes’ weight and length were measured. The percentage of the testicular weight was calculated relative to the body weight. The testicular volume was calculated as (width^2^ × length)/2, and was expressed as a percentage relative to the body weight. To perform the homogenates, the right testes were homogenized in 4 mL of PBS (pH 7.2) and centrifuged at 1200× *g*, for 20 min at 4 °C. Supernatants were collected and stored at −80 °C until the hormonal assays. The left testes and epididymis were fixed in a 10% buffered formalin solution (pH 7.4) for 24 h. Then, the samples were trimmed, embedded in paraffin wax, sectioned at 3 µm thickness, and stained with hematoxylin and eosin (H&E) for a light microscopic examination.

The content of the spermatozoa in the testes and epididymis was semi-quantitatively assessed and categorized according to the spermatozoa found in >75% of the epididymal ducts and testicular tubules as: 0 (an absence of spermatozoa), 1+ (few spermatozoa, where the lumens of the tubules were partially empty), and 2+ (the tubules were filled with abundant spermatozoa) [12,13]. For the measurement of the seminiferous tubule diameter, a total of 10 round cross sections of the seminiferous tubules were chosen in each rat. Then, two perpendicular diameters of each cross-section of the seminiferous tubules were measured at a magnification of ×400 and the mean of these was calculated. Moreover, the germinal epithelium height of the seminiferous tubules was also measured in four equidistant parts in each cross-section of the seminiferous tubules, and the mean was calculated [17].

Finally, the degree of testicular degeneration, characterized by few or no germ cells and sustentacular cells, was established as follows: 0 (an absence of testicular degeneration), 1+ (<25% of tubules affected), 2+ (25–50% of tubules affected), and +3 (>75% of tubules without germ cells).The results that are shown correspond to the 4th week of the experiment (the beginning of treatment) and the 20th week of the experiment (the end of treatment).

### 2.5. Sperm Quality

Epididymal sperms were collected immediately after euthanizing the animals. The right epididymis were incubated in Ham’s F10 medium and a thick cut on the tail of the epididymis was performed to allow the sperm to swim out of the epididymis [18]. The sperm motility was evaluated by placing a drop of sperm suspension between a slide and a cover slip and was observed at 100× using a phase contrast microscope. A total of four different fields were evaluated and expressed as a percentage of the motile sperm of the total sperm counted. The sperm counts were obtained as described by Badkoobeh et al. [18]. Briefly, 5 µL of the sperm suspension was fixed in a solution containing 0.35% of formalin, 5% NaHCO_3_, and 0.25% of trypan blue. Approximately 10 µL of the fixed sperm solution was transferred to a Neubauer chamber to perform cell counts in light microscopy at 400× magnifications. The sperm concentrations were expressed as millions per ml (10^6^/mL). To analyze the sperm viability, 20 µL of the sperm suspension was mixed with a stained solution containing 1% eosin Y and 5% nigrosine, and placed on microscope slides. Then, the slides were viewed in a light microscope at 400× magnifications [18]. Live sperm were not stained, and dead sperm were stained. In total, 200 spermatozoa were counted on each slide and the results were expressed as a percentage of the viable spermatozoa of the total sperm count. To assess the membrane integrity, a hypo-osmotic swelling test (HOST) was used, as previously described by Vaez et al. [19]. A total of 20 µL of the sperm suspension was mixed with 200 µL of the hypo-osmotic solution (100 mOsm/kg), consisted in 0.735 g of sodium citrate and 1 g of fructose, dissolved in 100 mL of distilled water, and the mixture was incubated at 37 °C over an hour. Then, a smear of the content was carried out and observed under a phase-contrast microscope at 400× magnifications. In total, two hundred spermatozoa per sample were counted. The spermatozoa with coiled tails were considered to be HOST-positive sperm. The results were expressed as a percentage of the HOST-positive sperm of the total sperm count.

### 2.6. Hormone Determinations

The serum and testes homogenates steroid hormone concentrations (estrone sulphate (SO4E1), 17b-estradiol (E2), androstenedione (A4), testosterone (T) and progesterone (P4)) were measured by a competitive enzyme immunoassay (EIA), previously validated by [9]. All the antibodies were developed in the Department of Animal Physiology (UCM, Madrid, Spain). Dihydrotestosterone (DHT) and dehydroepiandrosterone (DHEA) serum and the testes homogenates concentrations were measured by using a commercial enzyme immunoassay kit (Demeditec Diagnostics GmbH, Kiel, Germany), following the manufacturer’s instructions, and specific for this species.

Briefly, 96-well flat-bottom medium-binding polystyrene microplates (Greiner Bio-One, Madrid, Spain) were coated with the appropriate purified antibody dilution overnight at 4 °C. Afterward, the plates were washed and coated with standard and tumor homogenate samples that had been previously diluted in a conjugate working solution (CWS). After conjugate incubation, the plates were washed, and Enhanced K-Blue TMB substrate (Neogen, Lexington, KY, USA) was added to each well. Finally, colorimetric reaction was stopped by the addition of 10% H2SO4 to each well. The absorbance was read at 450 nm using an automatic plate reader. The hormone concentrations were calculated by the means of a software developed for this technique (ELISA AID, Eurogenetics, Brussels, Belgium). A standard dose–response curve was constructed by plotting the binding percent against each steroid hormone standard concentration. All the hormone concentrations were expressed in ng/gr for the testes homogenates, and in ng/mL for the serum samples, except the E1SO4 and E2 serum levels that were expressed in pg/mL.

### 2.7. Statistics

The statistical analysis was conducted with IBM SPSS Statistics 27 software (UCM, Madrid, Spain). The results were expressed as the means ± SE. The Kolmogorov–Smirnoff test was used to assess the goodness-of-fit distribution of the collected data. Most of the parameters that were studied were noted to be parametric (body weight, testicular volume and weight, sperm count and hormone integrity, histological parameters, and hormonal determinations). For the comparison between the control and experimental groups, the means were analyzed via one-way analysis of variance (ANOVA), followed by a Bonferroni post hoc test. The parameters of sperm motility and viability were noted to be non-parametric, therefore, the Kruskal–Wallis test was used to compare the data. For the comparison between the control and experimental groups, a Mann–Whitney test was used. In all the statistical comparisons, *p* < 0.05 denoted significant differences.

## 3. Results

### 3.1. Body and Testicular Weight

**Figure 1:**
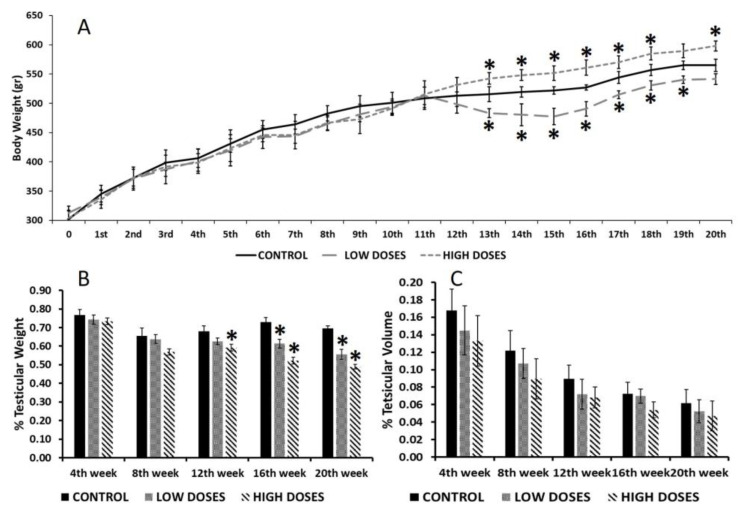
Body and testicular measurements during weeks of experimentation. (**A**) Body weight; (**B**) percentage of testicular weight; and (**C**) percentage of testicular volume, from control and experimental groups. Means were analyzed by ANOVA test followed by Bonferroni post hoc test. * denoted significant differences between control and experimental groups.

The results on body weight revealed that, during the first weeks of experimentation, the consumption of isoflavones at low or high doses did not affect body weight significantly. However, after the 12th week of experimentation, the group that were administered with low doses demonstrated a significant loss in body weight (*p* < 0.05) compared to the control group, while those receiving high doses showed a significant gain in body weight (*p* < 0.05) (Figure 1A). Nevertheless, no differences in the food and water consumption were observed among the three experimental groups (Figure S1).

In addition, testicular weight (Figure 1B) was significantly reduced (*p* < 0.05) in the rats administered with low and high doses of isoflavones from 12th week of experimentation. However, there were no significant differences in the testicular volume between the control and experimental groups (Figure 1C).

### 3.2. Testis Histopathological Analysis

A histopathological analysis of the testis of male rats (Table 1), revealed that the content of spermatozoa within the seminiferous tubules and epididymis was abundant in the control and low dose groups, and partially abundant in the high dose groups (80% of rats had a moderate content of spermatozoa, and 20% had an abundant content of spermatozoa) at the beginning of treatment. At the end of treatment, the low and control dose groups continued to have an abundant content of spermatozoa. Interestingly, in the high dose group, 40% of the rats had no spermatozoa within the testis and epididymis, and the rest had moderate or numerous spermatozoa. However, these differences between the control and high dose groups were not significant (*p* = 0.072).

**Figure 2:**
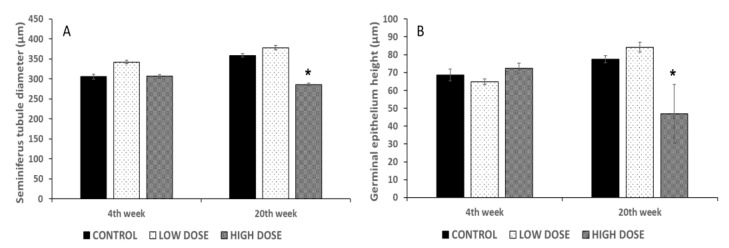
Effect of soy isoflavones in the seminiferous tubules diameter (μm) (**A**), and in the germinal epithelium height (μm) (**B**). Bars represent mean ± SD. Soy isoflavones significantly decreased the diameter of the seminiferous tubules and the germinal epithelium height in the high dose group at the end of treatment (20th week) compared to control group. No significant differences were found in the low dose group at the beginning and end of treatment compared to control group. * denoted significant differences between control and experimental groups (*p* < 0.05).

Regarding the seminiferous tubule diameter, the results showed that the administration of isoflavones at high doses caused a significant reduction (*p* < 0.05) in the tubular diameter compared to the control and low dose group at the end of treatment (Figure 2A). Likewise, the high dose group had a significant reduction (*p* < 0.05) in the germinal epithelium height at the end of treatment compared to the control group (Figure 2B). No significant differences were found between the control and experimental groups at the beginning of treatment.

**Figure 3:**
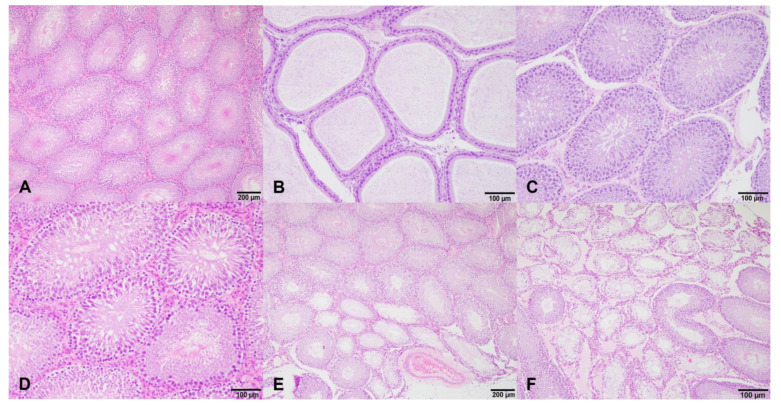
Histological sections of the testis and epididymis of male rats of control group (**A**,**D**) and treated groups (**B**,**C**,**E**,**F**) at different dosages of isoflavones. (**A**,**D**): Normal seminiferous tubules and normal Leydig cells; (**B**): presence of a normal sperm reserve in the epididymis of a rat treated with a low doses of isoflavones at the beginning of treatment; (**C**): testis of a rat treated with high doses of isoflavones at the beginning of treatment, with no histopathological changes in the germinal epithelium of seminiferous tubules and presence of mature spermatozoa in the lumen; and (**E**,**F**): testicular degeneration of rats treated with low (**E**) and high (**F**) doses of isoflavones at the end of treatment. Most seminiferous tubules and less than 25% of tubules were degenerated in low-dosage- and high-dosage-treated groups, respectively. Degenerated seminiferous tubules had thickness of the basement membranes and an absence of germinal epithelium, being lined exclusively by Sertoli cells (Sertoli cell pattern).

Differences were found in the testicular degeneration between the control and experimental groups at the end of treatment (Figure 3). In total, 40% of the rats from the low dose group and 80% of the cases from the high dose group presented with testicular degeneration. Specifically, for the high dose group, 40% of the rats with degeneration presented with degenerative changes in most of the seminiferous tubules of the testis, and in the other 40% of cases, in more than 25% of the tubules. At the beginning of treatment, a slight testicular degeneration, in less than 25% of the seminiferous tubules, was found in 20% of the rats from the low dose group.

The testicular degeneration of these cases was localized in the seminiferous tubules at the periphery of the tunica albuginea, except in those cases in which most of the seminiferous tubules were degenerated, where it was characterized by an increased thickness of the basement membranes and a total absence of germinal epithelium, being that the majority of the degenerated seminiferous tubules were exclusively lined by Sertoli cells (Sertoli cell pattern).

### 3.3. Sperm Quality

Our results revealed that the long-term consumption of high and low doses of isoflavones alters sperm quality (Table 2). From the start of treatment to the 16th week, there were no differences in the sperm quality. However, from the 16th week, the differences were notable, being statically significant (*p* < 0.05) by the end of the study. The sperm motility and sperm count were significantly reduced (*p* < 0.05) in the low and high dose groups compared to the control group. However, the sperm viability and membrane integrity were only significantly reduced in the high dose groups.

### 3.4. Serum Steroid Hormone Determinations

**Figure 4:**
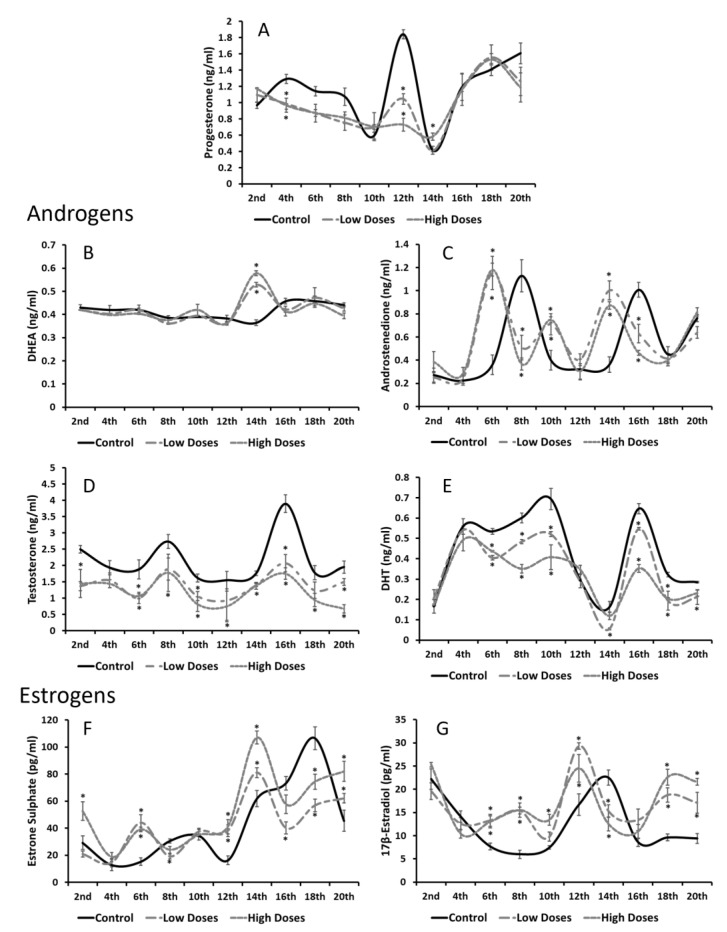
Changes detected in serum steroid hormone concentrations during weeks of isoflavone consumption. (**A**) Progesterone (P4) and androgens, as (**B**) dehydroepiandrosterone (DHEA) did not show significant alterations. (**C**) Androstenedione (A4); (**D**) testosterone (T); and (**E**) dihydrotestosterone (DHT) levels in experimental animals were reduced. However, estrogen levels as (**F**) estrone sulphate (E1SO4) and (**G**) 17β-estradiol (E2) were augmented compared to control group. Means were analyzed by ANOVA test followed by Bonferroni post hoc test. * denoted significant differences between control and experimental groups.

Regarding the serum steroid hormone concentrations, it can be observed that the control group presented physiological hormone fluctuations that were altered in the rats that were administered with the isoflavones, and that most of these fluctuations were related to estrogen and androgen levels (Figure 4). The P4 levels (Figure 4A) in the experimental and control groups follow the same pattern, denoting that the P4 concentrations diminished significantly (*p* < 0.05) with the low and high doses of the isoflavones during the first weeks of the experiment.

On behalf of the androgen concentrations, no significant differences were found in the DHEA levels, however, the A4 concentrations revealed alterations (Figure 4B,C). The control and experimental groups followed the same pattern: in the control group, two peaks on the serum A4 concentrations in the 8th and 16th weeks of the experiment can be observed. Nevertheless, for the low and high doses of the isoflavones, these two peaks correspond to the 6th and 14th weeks, 1 week before the control group, resulting in significant changes (*p* < 0.05) between the groups. Additionally, the T and DHT results showed that the isoflavone consumption at the low and high doses reduced these androgen levels significantly (*p* < 0.05) during all the experiments (Figure 4D,E).

In terms of the estrogen concentrations (Figure 4F,G), significantly higher levels of both the estrogens analyzed (E1SO4 and E2) were found in the experimental groups compared to the control group. The control group also showed an increase in the E1SO4 levels at the 16th week, and in the E2 in the 14th week. As for androgens, the rats with the high and low doses of the isoflavones presented the same increases before the control group did (in the 16th week for the E1SO4 concentrations, and in the 12th week for the E2 concentrations).

### 3.5. Testis Steroid Hormone Determinations

**Figure 5:**
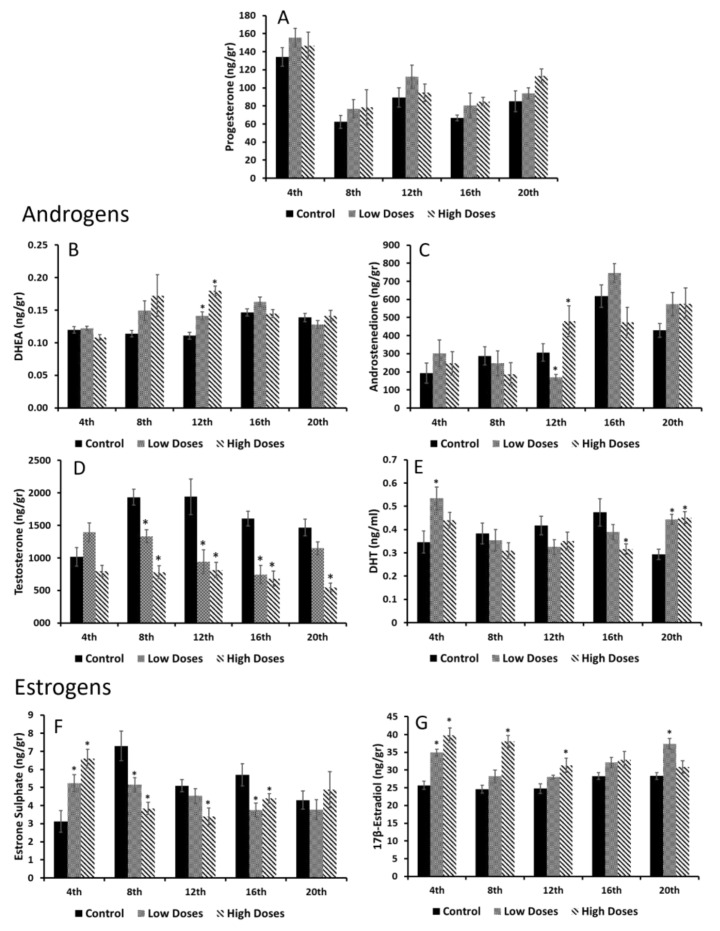
Testis homogenate hormone concentrations during weeks of isoflavones consumption. (**A**) Progesterone (P4); (**B**) dehydroepiandrosterone (DHEA); and (**C**) androstenedione (A4) did not show significant differences between control and experimental groups. (**D**) Testosterone (T) levels were reduced in experimental groups, but (**E**) dihydrotestosterone (DHT) was similar to control group. Moreover, as (**F**) estrone sulphate (E1SO4) levels were diminished in isoflavone groups, (**G**) 17β- estradiol (E2) tended to increase. Means were analyzed by ANOVA test followed by Bonferroni post hoc test * denoted significant differences (*p* < 0.05) between control and experimental groups.

Although in the serum hormone concentrations, several changes were observed in the isoflavone groups, in the testis hormone concentrations, the differences that were found were not that significant, except for the androgen and estrogen levels (Figure 5). Regarding the P4, DHEA, and A4 (Figure 5A–C), no significant differences were found between the control and experimental groups.

Interestingly, the testis T levels were reduced in the low and high mixtures of the isoflavones compared to the control group (Figure 5D). This reduction was significant (*p* < 0.05) from the 8th week until the end of experiment. In addition, the DHT concentrations (Figure 5E) tended to decrease, but not significantly from the 8th to 16th week of the experiment. However, in the 4th and 20th weeks, the DHT concentrations of the isoflavone groups were significantly increased (*p* < 0.05) compared to the control group.

On behalf of the testis estrogen levels, changes were found between the control and experimental groups (Figure 5F,G). At the 4th week of experiment, a significant increase (*p* < 0.05) was observed in the E1SO4 levels of the rats undergoing isoflavone consumption, although from the 8th week, these levels were significantly decreased (*p* < 0.05) until the end of experiment. However, the E2 levels in the isoflavone groups were significantly higher (*p* < 0.05) from the 4th week until the 20th week.

**Figure 6:**
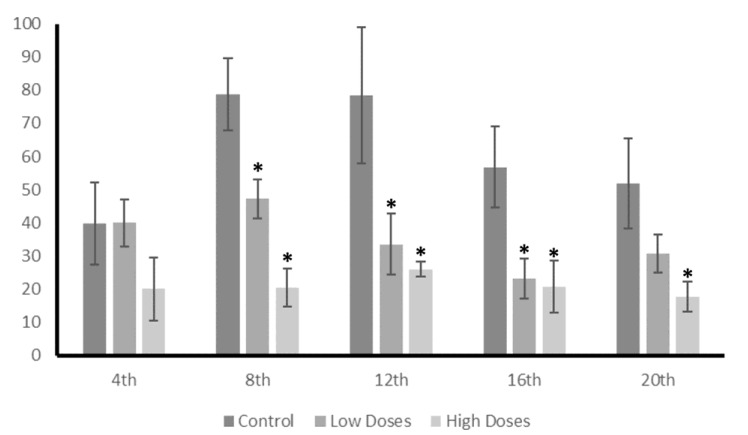
Testis T/E2 ratio. Long exposure to low and high doses of isoflavones produced a hormonal imbalance leads to a reduction in T/E2 ratio. Data were analyzed by ANOVA test followed by Bonferroni post hoc test. * denoted significant differences (*p* < 0.05) between control and experimental groups.

These differences in the T and E2 concentrations between the control and experimental groups are also reflected in the T/E2 ratio (Figure 6). The results showed a reduction in the T/E2 ratio of the isoflavone groups from the 8th week until the end of experiment, denoting a hormonal imbalance.

## 4. Discussion

Extensive studies have contemplated the beneficial effects of the consumption of isoflavones on the relief of menopausal symptoms, lowering the risk of cancer, or decreasing the risk of obesity [3,20]. However, different investigations have elucidated that their intake can cause certain disorders, especially hormonal, that affect the reproductive tract [9,21].

Nevertheless, the effect, beneficial or detrimental, of isoflavones depends on multiple factors, such as age, sex, the dose ingested, or the time of the intake. As they are compounds that are structurally similar to 17b-estradiol [20], it has been observed that, during critical periods of development, the intake of isoflavones causes hormonal disorders that can even delay the onset of puberty [9]. Therefore, in this study, the effect of a long exposure to isoflavones on the reproductive function in adult male rats is determined.

Isoflavones and obesity have been linked due to the estrogenic characteristic of isoflavones. It has been speculated that isoflavones reduce body weight [3,22] by decreasing the activity of the lipoprotein lipase (LPL) [23]. This is partially explained by E2 playing an important role in the regulation of adipocyte development [24]. When E2 binds to the estrogen receptors, they decrease the LPL activity and, therefore, lipogenesis [23]. Interestingly, in vitro results on mouse bone marrow cells have showed that a low exposure to genistein inhibited adipogenesis, whereas higher levels of genistein produced a stimulation of adipogenesis [25]. According to this, our study revealed that the intake of low concentrations of isoflavones produced a significant loss of body weight in male rats from the 12th week of intake. However, exposure to higher concentrations produced a significant gain in body weight. In vivo studies with rats have also revealed controversial results. Some studies have reported that the intake of high doses of genistein did not affect body weight [22], whereas other studies have demonstrated that dietary isoflavones decrease body weight [5]. This discrepancy of results could be due to the time of the exposure and the animal gender. Most of the studies were carried out on female rodents, whose metabolism is different to males, as this study demonstrated. Additionally, in this study, the differences in body weight started to be notable 12 weeks after the beginning of the experiment, therefore, the animals were consuming dietary isoflavones for a long time before observing the effects, denoting an accumulative impact of the isoflavones on males.

Isoflavones also affect testicular weight. Our results revealed that a long exposure to dietary isoflavones produces a significant decrease in the testis weight/body weight ratio of adult male rats. Preliminary studies using prepuberal male rats did not show differences in their testis weights after a short-term exposure to isoflavones [12], however, other studies have shown that different isoflavone diets produced an increase in testis weight [26].

Therefore, the differences found in the testis weights could affect the reproductive function. As endocrine disruptors, isoflavones can affect testicular structures and dysregulate the spermatogenesis processes that lead to the production of deficient sperm [14,27]. The histology results from the control and experimental rats revealed the presence of abundant spermatozoa in the seminiferous tubules in all groups, denoting that spermatogenesis would not be affected by isoflavones administration. However, the results obtained in the study of the sperm quality parameters (the sperm count, sperm viability, sperm motility, and membrane integrity) showed significant reductions in the rats administered with high doses of isoflavones compared to the control group. Interestingly, in the low dose groups, only the sperm motility and sperm count were significantly reduced compared to the control group. According to this, several studies have demonstrated that a long-term exposure to phytoestrogens affects reproductive success by reducing sperm quality parameters such as production and motility [28]. Indeed, it has been suggested that phytoestrogens can be present in the reproductive system, influencing spermatogenesis and affecting the sperm quality in Chinese men [10]. Therefore, a continuous exposure to isoflavones in low and high doses affects sperm quality, which can lead to problems in fecundity [29]. These changes found in the sperm quality parameters could be related to the reduction in the diameter of the seminiferous tubules and the germinal epithelium height. Significant differences in these measures were found in the high dose groups, in which the sperm quality parameters were reduced as well. Preliminary studies revealed that the consumption of isoflavones during puberty affects the number of spermatozoa. Low doses of isoflavones caused a great reduction in the content of spermatozoa in the seminiferous tubules, whereas the rats treated with high doses of isoflavones showed a fewer number of spermatozoa [13]. These results corroborate that isoflavones can exert an effect on the last stages of spermatogenesis processes, by altering the hormonal components and reducing the quantity of the available spermatozoa. Other authors have also demonstrated that the consumption of isoflavones causes a reduction in the seminiferous tubule diameter and germinal epithelium height [17]. Spermatogenesis occurs in the germinal epithelium and is controlled by hormones and other factors. Leydig and Sertoli cells are responsible for producing androgens and estrogens, which regulate the spermatogenesis process [2]. Therefore, an imbalance in the androgen or estrogen production can affect this process [2,17,30].

Our results revealed a hormonal imbalance in the serum and testis steroid hormone levels. It can clearly be noticed that the serum androgens (T and DHT) were significantly decreased, whereas the serum estrogens (E1SO4 and E2) were significantly increased in the experimental groups compared to the control. These results are in line with other authors that have observed that the consumption of isoflavones significantly reduces the androgen circulating levels in males, despite the developing period and time of consumption [7,9]. However, the effects of isoflavones on the circulating estrogen levels are controversial. Our study showed an increase in the estrogen levels in the animals exposed to the isoflavones; according to this, the preliminary studies developed in our laboratory also revealed an increase in these circulating estrogen levels after the administration of isoflavones during puberty [9], the same as other authors that have shown similar results [31,32]. Nevertheless, certain authors did not obtain any differences of the serum E2 concentrations after a short-term exposure to isoflavones, during three developing periods [7]. Despite this, isoflavones exert an effect on hormonal secretion that can trigger dysfunctions in the target organs of these hormones.

It is known that hormonal homeostasis shows periodic fluctuations in order to maintain an internal balance, and gonadal steroids are known to exhibit these circadian oscillations [33]. Interestingly, all the circulating hormones that were determined in this study showed different maximum concentrations at certain times. The A4, T, and E1SO4 serum concentrations revealed two maximum peaks of their concentrations around the 8th and 16th weeks of the experiment in the control animals, whereas hormones such as E2 or P4 showed only one maximum peak of concentration throughout the weeks of experiment. These results prove that gonadal steroids not only showed daily oscillations, but also monthly oscillations, which regulate the hormonal homeostasis. Beyond these results, the animals that were exposed to isoflavones at low or high concentrations exhibited the same A4 and E1SO4 maximum peaks of concentrations, but weeks earlier than the control group. It is demonstrated that low doses of isoflavones delay the onset of puberty in male rats [9,12]. In this preliminary study, the maximum peak of the circulating T that indicates the onset of puberty in male rats was shown 1 week before that in the rats fed with low doses of isoflavones, denoting that isoflavones alter gonadal secretion and exert an effect on development. In the present study, it is notable that the exposure to isoflavones accelerated the natural secretion hormonal oscillations that can cause an acceleration of developmental processes. Recent studies have shown an association between a decrease in the serum T levels and aging [34]. Considering that isoflavones decrease the serum T levels and accelerate the hormonal oscillations, it can be hypothesized that a long exposure to isoflavones from adulthood can accelerate the aging process in males, although further studies are needed. On the contrary, Al-shaikh, [34] revealed that the consumption of isoflavones during aging stages (elder rats) may protect against age changes in the testis. Therefore, the effects of isoflavones may depend on the developmental window in which they are consumed. In males, the production of sex steroid hormones occurs mainly in the testis, thus, the differences found in the secreted levels indicate that there must be alterations in the production of these hormones in the testis. These organs are sensitive to hormonal changes, and it is known that isoflavones are capable of reaching the reproductive organs [7,35], leading to alterations in sex hormone production and secretion. In this study, several changes in the testis hormone production have been found. The results showed a slight, but not significant, increase in the concentrations of sex steroid precursors (P4, DHEA, and A4) in the rats exposed to isoflavones, compared to the control. These precursors are present in Leydig cells and are involved in the production of the estrogens and androgens necessary for the spermatogenesis process [9,14,15]. Increasing the concentrations of the precursors might indicate that the sources of the active estrogens and/or androgens are deficient, thus, there is a need to obtain substrates to produce the sex steroid hormones and, therefore, maintain the hormonal homeostasis.

In this study, a deficiency in androgen synthesis was observed either in the T or DHT levels in the testis homogenates, which decrease in the experimental groups compared to the control. It has been demonstrated that, during developmental periods, isoflavones decrease the T testis concentrations, leading to disorders in reproductive function [9,36]. This decrease in the T testis levels can be related to the sperm quality deficiency found in these animals, since T is crucial for the spermatogenesis process. Zhu et al. [36] also observed that isoflavones affect the T synthesis in Leydig cells, which also leads to a dysfunction in the Sertoli cells. This decrease in the T levels of the rats fed with the isoflavones is due to the action of isoflavones on the testis that augment the E2 levels, as shown in the results. Indeed, the results showed that the E1SO4 testicular concentrations decrease significantly in the experimental animals compared to the control. E1SO4 is considered to be an inactive estrogen. The organism sulfoconjugates estrogens in order to inactivate them through the enzyme estrogen sulfotransferase. This enzyme is expressed in the testis and regulates the local exposure to estrogens, protecting the testis against excess estrogen concentrations [37,38]. Therefore, these results denoted that isoflavones influence the testis estrogen levels by preventing their inactivation and promoting an estrogenic testis microenvironment that is adverse for a correct spermatogenesis process.

These changes in the levels of the androgens and estrogens that were observed in the testis of the male rats treated with the isoflavones, caused a variation in the T/E2 ratio, which is essential for correct testicular function. In this study, a significant decrease in the T/E2 ratio was shown during the weeks of the experiment. The T/E2 ratio is widely used in order to provide information regarding the testis functionality, and has been proposed as a metric for determining sexual dysfunctions [39,40,41] or the risk of developing cerebrovascular diseases [42]. Therefore, this study determined that isoflavones cause a decrease in T/E2, which compromises the sperm quality and testis functionality in adult male rats.

## 5. Conclusions

This study demonstrates that the effect of isoflavones depends on the amount of isoflavone intake, in addition to the time of the intake. The results obtained on male rats administered with high doses of isoflavones show greater reductions in reproductive function than that in those administered with a regular intake of isoflavones (low doses). However, in research, the use of doses higher than the normal intake level is required when the lower doses do not provide optimal results, but their effects must be taken into account when translated to humans.

Altogether, these results suggest that continuous exposure to isoflavones in adult male rats causes a hormonal imbalance in the testes, maintaining an estrogenic environment that leads to a disruption in the production and secretion of androgens and estrogens. This alteration in hormonal homeostasis leads to histological changes, such as a reduction in the seminiferous tubules’ diameter and the germinal epithelium height. These changes result in defects in the spermatogenesis processes that will lead to a reduction in the sperm quality.

In conclusion, the long-term consumption of low and high doses of isoflavones in adult male rats compromises their testicular functionality, by causing an imbalance in hormonal homeostasis that leads to a reduction in the sperm quality.

## Figures and Tables

**Table 1:** Percentage of the content of spermatozoa (SC) in the caudal epididymis at 4th week (beginning of treatment) and 20th week (end of treatment), categorized according to the spermatozoa found in >75% of the epididymal tubules as: 0 (absence of spermatozoa), 1 (few spermatozoa) and 2 (abundant content of spermatozoa).

Testicular Content of Spermatozoa						
	4th week			20th week		
	0	1	2	0	1	2
Control	0	0	100	0	0	100
Low doses	0	0	100	0	0	100
High doses	0	80	20	40	20	40

**Table 2:** Results from sperm quality analyses (sperm motility, count, viability, and membrane integrity) in control and experimental groups at the end of experiment.

	Sperm Motility (% Motile Sperm)	Sperm Count (×10^6^ Sperm/mL)	Sperm Viability (% Live Sperm)	Membrane Integrity (% HOST Positive)
Control	86.67 ± 11.54	21.70 ± 2.56	91.80 ± 4.08	73.4 ± 4.10
Low Doses	62.53 ± 6.41 *	11.10 ± 1.50 *	86.20 ± 8.31	69.31 ± 1.12
High Doses	40.17 ± 5.17 *	3.28 ± 0.80 *	51.20 ± 6.08 *	57.44 ± 0.59 *

* Denoted significant differences between control and experimental groups.

## Data Availability

The data that support the findings of this study are available from the corresponding author upon reasonable request.

## Supplementary Materials

The following supporting information can be downloaded at: https://www.mdpi.com/article/10.3390/nu15051261/s1, Figure S1: Absolute food (g/rat/week) and water (ml/rat/week) consumption during the weeks of experiment.
