# Supplementary figures and images for: Long-Term Exposure to Isoflavones Alters the Hormonal Steroid Homeostasis-Impairing Reproductive Function in Adult Male Wistar Rats
Source: Nutrients. 2023 Mar 2;15(5):1261. doi: 10.3390/nu15051261 (PMC10005734; doi:10.3390/nu15051261)

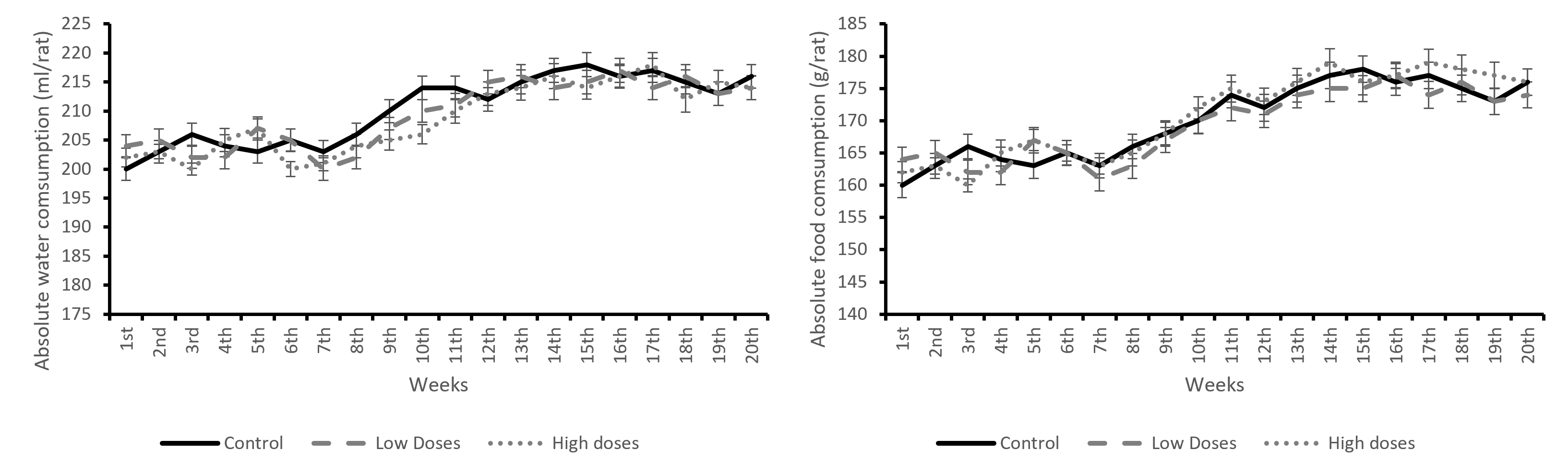

Supplement: Supplementary file 1 [file nutrients-15-01261-s001.zip › nutrients-2230333-supplementary.tif]
